# Psychosocial factors impacting barriers and motivators to cancer genetic testing

**DOI:** 10.1002/cam4.5709

**Published:** 2023-02-19

**Authors:** Erika N. Hanson, Emerson Delacroix, Sarah Austin, Grant Carr, Kelley M. Kidwell, Elizabeth Bacon, Lynette Hammond Gerido, Jennifer J. Griggs, Elena M. Stoffel, Ken Resnicow

**Affiliations:** ^1^ Department of Internal Medicine University of Michigan Ann Arbor Michigan USA; ^2^ School of Public Health, Department of Health Behavior and Education University of Michigan Ann Arbor Michigan USA; ^3^ School of Public Health, Department of Biostatistics University of Michigan Ann Arbor Michigan USA; ^4^ Center for Health Communications Research, Rogel Cancer Center, Michigan Medicine Ann Arbor Michigan USA; ^5^ Department of Medicine, Division of Hematology/Oncology University of Michigan Ann Arbor Michigan USA; ^6^ School of Public Health, Department of Health Management and Policy University of Michigan Ann Arbor Michigan USA; ^7^ University of Michigan, Institute for Healthcare Policy and Innovation Ann Arbor Michigan USA

**Keywords:** barriers, genetic testing, hereditary cancer, motivators

## Abstract

**Background:**

Only a small proportion of patients who qualify for clinical genetic testing for cancer susceptibility get testing. Many patient‐level barriers contribute to low uptake. In this study, we examined self‐reported patient barriers and motivators for cancer genetic testing.

**Methods:**

A survey comprised of both new and existing measures related to barriers and motivators to genetic testing was emailed to patients with a diagnosis of cancer at a large academic medical center. Patients who self‐reported receiving a genetic test were included in these analyses (*n* = 376). Responses about emotions following testing as well as barriers and motivators prior to getting testing were examined. Group differences in barriers and motivators by patient demographic characteristics were examined.

**Results:**

Being assigned female at birth was associated with increased emotional, insurance, and family concerns as well as increased health benefits compared to patients assigned male at birth. Younger respondents had significantly higher emotional and family concerns compared to older respondents. Recently diagnosed respondents expressed fewer concerns about insurance implications and emotional concerns. Those with a BRCA‐related cancer had higher scores on social and interpersonal concerns scale than those with other cancers. Participants with higher depression scores indicated increased emotional, social and interpersonal, and family concerns.

**Conclusions:**

Self‐reported depression emerged as the most consistent factor influencing report of barriers to genetic testing. By incorporating mental health resources into clinical practice, oncologists may better identify those patients who might need more assistance following through with a referral for genetic testing and the response afterwards.

## Abstract 2

Many factors influence an individual's barriers and motivators to getting cancer genetic testing including age, sex, having children, time since cancer diagnosis, cancer type, and mental health. Depression showed the largest impact: those with higher depression scores were significantly more likely to endorse more barriers to testing.

## 1 INTRODUCTION

An estimated 5%–10% of all cancers have a hereditary cause yet many patients who meet National Comprehensive Cancer Network (NCCN) guidelines for clinical genetic testing for cancer susceptibility do not undergo testing. Guidelines suggest testing is indicated for all patients with pancreatic adenocarcinoma, however, only around 20% of patients get tested.^1^ Patients with prostate cancer have a low rate of germline genetic testing uptake with one study finding that only 11% of the eligible patients completed testing.^2^ Fewer than 20% of breast and ovarian cancer patients who qualify for testing based on NCCN guidelines receive testing.^3^, ^4^ Another study found that among women with breast cancer who qualify for genetic testing per NCCN guidelines, only about 60% were referred and about 46% completed testing indicating that despite a referral, there is still a reduction of uptake in germline testing.^5^ Patients at risk for Lynch Syndrome diagnosed with either colorectal or endometrial cancer have even lower levels of testing with one study reporting a rate around 6% and another study reporting a rate around 3%.^6^, ^7^ Germline genetic testing for patients with cancer can have significant impact on their cancer treatment and surveillance as well as implications for family members.

Reasons for low uptake of genetic testing can be broken down into patient, provider, and systemic barriers. Patient‐level factors, defined as factors impacting or perceived by an individual patient, include lack of provider referral and concerns about cost, clinical utility, privacy and confidentiality, and logistical issues.^8^, ^9^, ^10^, ^11^ The provider reported barriers to testing include perceived patient disinterest, long wait times for genetic services, insurance coverage concerns, unknown guidelines for management, insufficient experience/training, and clinical time limitations.^12^, ^13^, ^14^, ^15^ Systemic barriers include reduced access to specialists for under‐ and uninsured patients, health system mistrust, and lack of pre‐symptomatic genetic testing with public insurance.^16^, ^17^, ^18^ Although there is suboptimal utilization of genetic testing across all cancer types, much of the current literature regarding the barriers to genetic testing focuses on patients with breast and ovarian cancer. Less is known about barriers among patients with other cancer types. Additionally, there is a gap in the literature investigating patient‐level motivators for genetic testing.

In this study, we aimed to identify patient‐level barriers and motivators to genetic testing across multiple cancer types. Within the barrier and motivator domains, we created empirically derived subscales for emotional concerns, insurance concerns, social and interpersonal concerns, family worry, internally motivated emotions, health benefits, and interpersonal emotions. We hypothesized that sex, age, having children, and diagnosis of a cancer type associated with hereditary cancer syndromes would be associated with an increase in specific barriers to testing. Specifically, we hypothesized that (1) those assigned female at birth would have increased family concerns and social and interpersonal concerns based on prior literature^19^, ^20^, ^21^; (2) younger respondents would have increased scores on all barrier subscales and the increased health benefits motivator subscale as cancer is often regarded as a disease related to older age^21^, ^22^, ^23^; (3) having children would be associated with increased scores on the family concern subscale; (4) having a diagnosis of a BRCA‐associated cancer type would be associated with higher scores on the social and interpersonal concerns subscale and the health benefits subscale due to the prevalence of information regarding BRCA 1/2 testing implications.^24^

## 2 METHODS

### 2.1 Study design and cohort selection

This cross‐sectional study was conducted to better understand barriers and drivers for genetic testing and ultimately inform an intervention being deployed a as part of a larger randomized trial. We queried the University of Michigan patient registry to identify individuals with a documented in‐patient or out‐patient encounter at Michigan Medicine between January 1, 2019, and February 15, 2021, with an ICD‐9/10 code for cancer with one of the following characteristics: diagnosed under the age of 50 for breast or prostate cancer, or any age for colorectal, endometrial, ovarian, pancreatic, or metastatic prostate cancer. These eligibility criteria were selected for patients who likely qualified for genetic testing by NCCN guidelines. Participants were shown a list of 21 cancer types (bladder, bone, breast, cervical, colon, endometrial, head and neck, Hodgkin's lymphoma, leukemia, liver, lung, melanoma, non‐Hodgkin's lymphoma, oral, ovarian, pancreatic, prostate, rectal, renal, non‐melanoma skin, and stomach) and selected all primary cancer types that they had been diagnosed with in the past. Medical chart review was not performed to confirm diagnoses. Of the 376 respondents, 8 (2.1%) did not report one of the six original cancers but were included in the analysis as they did report a diagnosis of cancer, received genetic testing despite a lack of NCCN guidelines, and we felt that their psychosocial barriers and motivators would align with the rest of the group.

### 2.2 Ethical considerations

The University of Michigan Medical School Institutional Review Board (IRB) approved this study, which involved contacting patients who met the eligibility criteria via email (HUM0019157). Potential participants were presented with a consent and waiver of formal written consent was granted by the IRB.

### 2.3 Measures

Demographic variables collected include sex assigned at birth, age, race/ethnicity, education, income level, insurance status, number of biological children, and employment status. Patients were asked to report all primary cancer types, date of cancer diagnosis, treatment received, and genetic testing status. The PHQ‐4 was used to measure anxiety and depression; respondents scores were dichotomized by either none (0–2) or mild to severe (3–12).^25^ As part of the survey, participants were asked if they had undergone cancer genetic testing. For this analysis, only participants who had reported receiving germline cancer genetic testing were included.

We gathered individual questions from existing measures of knowledge, attitudes, and perceptions of barriers and benefits of genetic testing, adding new items where needed.^11^, ^14^, ^26^, ^27^, ^28^, ^29^, ^30^, ^31^ Barrier items in the survey encompassed several domains including worry, fear, limited genetics knowledge, cost, impact of testing, psychosocial distress, genetic discrimination, stigma, religious beliefs, and logistics such as healthcare utilization. Motivators included emotional impact and actionable knowledge leading to changes in healthcare. As testing was already completed, participants were asked to recall how true 33 barriers and nine motivators were to them before they had genetic testing using a 5‐point Likert scale (1 = not true at all, 5 = very true). Emotional responses after genetic testing were assessed using the validated, 12‐item Feelings About genomic Testing Results (FACToR) questionnaire (per subscale Cronbach's α 0.66, 0.78, 0.72, 0.70) which asks respondents to recall how they felt in the past week using a 6‐point Likert scale (1 = Not at all, 6 = A great deal).^32^

### 2.4 Study administration

An invitation to the study was sent by email with link to a Qualtrics survey was sent to 3000 potentially eligible individuals who were offered $10 upon completion. No reminders were sent. We targeted a sample of 350 respondents who had completed genetic testing to allow for subgroup comparisons.

### 2.5 Statistical analyses

Each domain‐ barrier, motivator, and FACToR‐ was analyzed separately to ensure the largest possible sample size for each analysis. For each set of measures (barriers, motivators, and FACToR), half of the data (split randomly) was used for an exploratory factor analysis (EFA), with the other half used for a confirmatory factor analysis (CFA). In EFA, parallel analysis was used to determine the number of latent factors present in the data, and factors were computed using principal axis factoring with a promax rotation to allow for correlated factors while maintaining a simple structure. The fit of the models identified in EFA were assessed in CFA models on the other half of the data. CFA models were fit using diagonally weighted least squares estimation with robust standard errors. CFA model fit was evaluated by considering comparative fit index (CFI), Tucker–Lewis index (TLI), root mean square error approximation (RMSEA), and the chi‐squared test statistic. Multiple indicators multiple causes (MIMIC) models were used to assess the effects of demographics and patient characteristics on the latent factors. Effects were considered statistically significant at the 0.05 level after controlling for multiple‐testing with the Benjamini–Hochberg correction. The “lavaan” and “psych” packages in R were used to analyze the data.

For this analysis, BRCA‐related cancer types included breast, ovarian, prostate, pancreatic, and melanoma, and Lynch‐related cancer types included colorectal, endometrial, stomach, ovarian, pancreatic, bladder, liver, and kidney. Respondents could fall into both BRCA‐ and Lynch‐related cancer type categories based on their cancer history. These cancer cluster syndromes were chosen because they are widely recommended for testing by clinicians and are most familiar to patients. Lynch was also chosen as it is not confounded by sex, as both males and females were found in the Lynch syndrome group.

## 3 RESULTS

**FIGURE 1:**
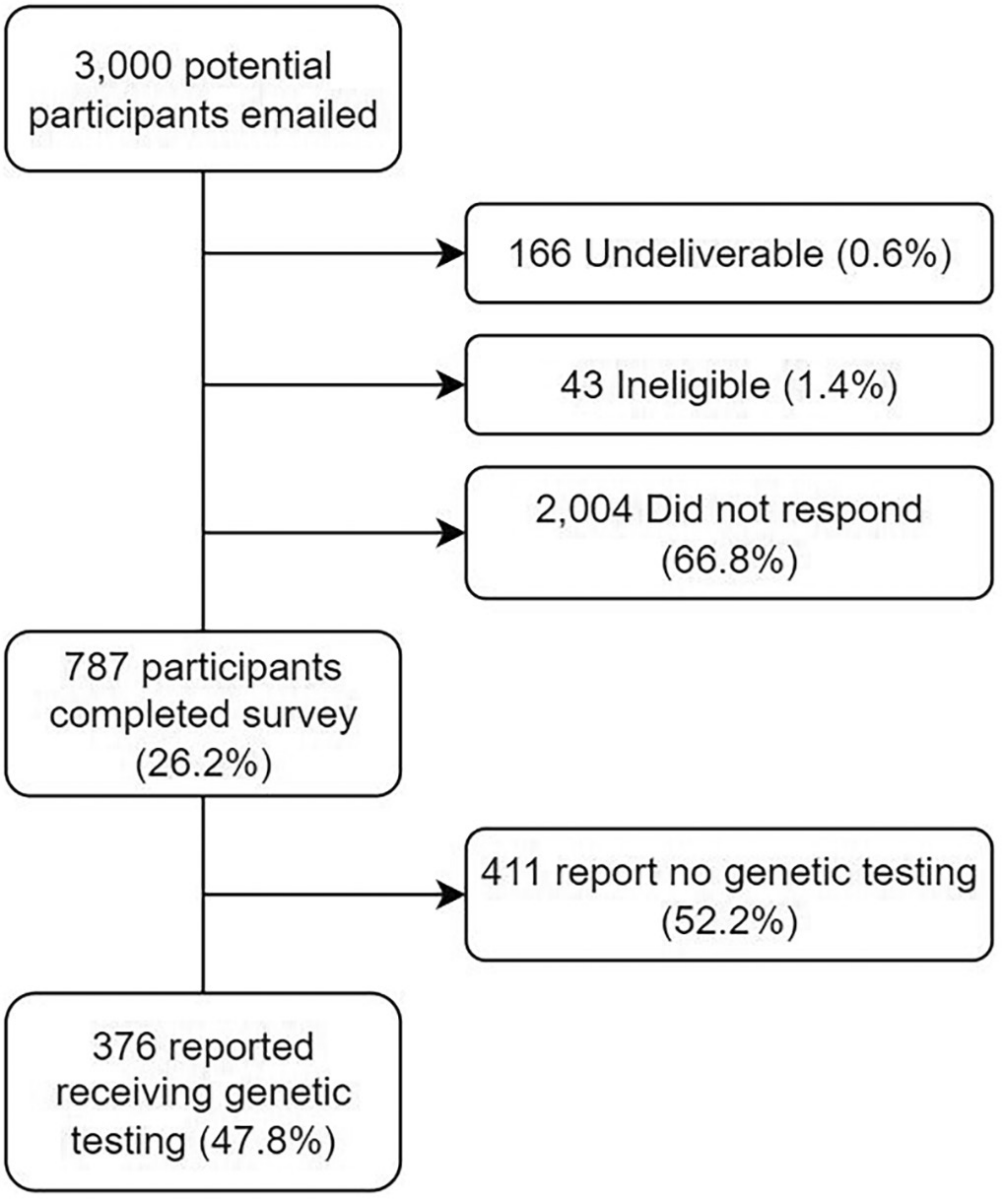
Participant flow diagram

Of the 3000 potential participants invited via email 166 (0.6%) were undeliverable, 43 (1.4%) were ineligible mostly due to not having a diagnosis of cancer, and 2004 (66.8%) did not respond (Figure 1). Out of the 787 participants who responded, 47.8% (*n* = 376) reported having had genetic testing and were included in our analysis. Most of these tested participants were assigned female at birth (*n* = 347/376, 92.3%). Age ranged from 21 to 85 years old (median age of 48 years). Years since cancer diagnosis was distributed across ≤2 (*n* = 94/376, 25.0%), 2–5 (*n* = 152/376, 40.4%), 5–10 (*n* = 88/376, 23.4%), and > 10 (*n* = 42/376, 11.2%). The most common cancer diagnosis was breast (*n* = 235) followed by ovarian (*n* = 79), pancreatic (*n* = 25), colorectal (*n* = 25), endometrial (*n* = 15), and prostate (*n* = 10). A majority of respondents reported having biological children (*n* = 289/376, 76.9%). Only 6.1% (*n* = 22/376) respondents reported anxiety and 11.2% (*n* = 40/376) reported depression as assessed by the PHQ‐4. Other demographics of the cohort are reported in Table 1.

**TABLE 1:** Demographics

	*N*	%
Sex at birth		
Female	347	92.3
Male	29	7.7
Race		
American Indian or Alaska Native	7	1.9
Asian or Asian‐American	20	5.3
Black or African American	11	2.9
Middle Eastern or North African	9	2.4
White, non‐Hispanic	338	90.1
Missing	1	0.3
Age		
≤ 45	139	37.1
46–65	184	49.1
≥ 66	52	13.9
Missing	1	0.3
Education		
Less than bachelors	96	25.5
Bachelors	131	34.8
Advanced degree	149	39.6
Income		
< $30,000	24	7.3
$30,000–$74,999	88	26.7
≥ $75,000	218	66.1
Missing	46	12.2
Health insurance		
Public/government	79	21.0
Private	291	77.4
Other sources	4	1.1
Missing	2	0.5
Years since cancer diagnosis		
≤ 2	94	25.0
2–5	152	40.4
5–10	88	23.4
> 10	42	11.2
BRCA 1 / 2‐related cancer		
No	41	10.9
Yes	335	89.1
Lynch syndrome‐related cancer		
No	266	70.3
Yes	110	29.3
Have children		
No	87	23.1
Yes	289	76.9
Anxiety		
No	336	93.9
Yes	22	6.1
Missing	18	4.8
Depression		
No	318	88.8
Yes	40	11.2
Missing	18	4.8

Results of the CFA for the three a priori domains – barriers, motivators, and FACToR questions can be found in Table 2. The barrier domain items best fit with a four‐factor solution (CFI = 0.93, TLI = 0.92) which we labeled emotional concerns (four items), insurance concerns (three items), social and interpersonal concerns (six items), and family concerns (three items). Emotional concerns include feeling scared, angry, hopeless, and anxious. Insurance concerns related to both life and health insurance. Social and interpersonal concerns related to feeling different or “other” when compared to the people around them. Family concerns, the fourth scale, assessed concerns over potential for family members developing cancer or finding pathogenic variants.

**TABLE 2:** Survey domains and scales

Retrospective barriers domains		
CFI = 0.93, TLI = 0.92, RMSEA = 0.061, *χ* ^2^ = 164.11, *p* < 0.001		
*Factor 1*	*Emotional concerns*	
	I thought if I were found to carry an altered gene, I worried I would feel … scared	
	I thought if I were found to carry an altered gene, I worried I would feel … angry	
	I thought if I were found to carry an altered gene, I worried I would feel … hopeless	
	I thought if I were found to carry an altered gene, I worried I would feel … anxious	
*Factor 2*	*Insurance concerns*	
	I worried about how it would affect my health insurance.	
	I worried it would be considered a pre‐existing condition for health insurance if I were found to carry an altered gene.	
	I worried it would affect my life insurance policy if I were found to carry an altered gene.	
*Factor 3*	*Social and interpersonal concerns*	
	I thought that if I carried an altered gene, it would cause me to feel less healthy than other people.	
	I thought that if I carried an altered gene, it would cause others to view me negatively.	
	I worried that if I carried an altered gene, I would feel singled out.	
	I was concerned about my partner's reaction to my genetic testing results.	
	I was concerned about my family's reaction to my genetic testing results.	
	I thought if I were found to carry an altered gene, I worried I would feel … ashamed	
*Factor 4*	*Family concerns*	
	I thought that if I carried an altered gene, it would cause me to worry more about other family members who could be carriers.	
	I thought that if I carried an altered gene, I would feel guilty if my family member developed cancer.	
	I would feel guilty if one of my relatives had an altered gene and I did not.	
**Retroactive motivator domains** CFI = 0.94, TLI = 0.91, RMSEA = 0.065, *χ* ^2^ = 42.36, *p* = 0.012		
*Factor 1*	*Internally motivating emotions*	
	I thought if I was found to carry an altered gene, I would feel … Prepared	
	I thought if I was found to carry an altered gene, I would feel … Knowledgeable	
	I thought if I was found to carry an altered gene, I would feel … Relieved to know	
	I thought if I was found to carry an altered gene, I would feel … Empowered	
*Factor 2*	*Health benefits*	
	Knowing my genetic status could help me plan my treatment.	
	Knowing whether or not I carry an altered gene would increase my sense of personal control.	
	Knowing that I carry an altered gene would help me decide whether to go for more frequent cancer screening.	
*Factor 3*	*Interpersonal emotions*	
	I thought if I was found to carry an altered gene, I would feel … Responsible	
	I thought if I was found to carry an altered gene, I would feel … Purposeful	
**FACToR domains** CFI = 0.78, TLI = 0.68, RMSEA = 0.096, *χ* ^2^ = 98.88, *p* < 0.001		
*Factor 1*	*Negativity/uncertainty*	
	How upset did you feel about your genetic test result?	
	How anxious or nervous did you feel about your genetic test result?	
	How sad did you feel about your genetic test result?	
	How frustrated did you feel that there are no definite disease prevention guidelines for you?	
	How uncertain did you feel about your genetic test result?	
	How uncertain did you feel about what your genetic test result means for your child(ren) and/or family's risk of disease?	
*Factor 2*	*Positive emotion*	
	How happy did you feel about your genetic test result?	
	How relieved did you feel about your genetic test result?	
*Factor 3*	*Positive outlook*	
	How much did you feel that you understood clearly your choices for disease prevention or early detection?	
	How helpful was the information you received from your genetic test result in planning for the future?	
*Factor 4*	*Privacy*	
	How concerned did you feel that your genetic test result would affect your health insurance status?	
	How concerned did you feel that your genetic test result would affect your employment status?	

For the motivator domain, the CFA yielded three factors (CFI = 0.94, TLI = 0.91) which we named internally motivating emotions (four items), health benefits (three items), and interpersonal emotions (two items). Internally motivating emotions assessed confidence‐inducing and uplifting emotions such as feeling prepared, knowledgeable, relieved to know, and empowered. The health benefit items assessed the impact of knowing genetic status when making healthcare decisions. Interpersonal emotions, the third scale, assessed how testing may increase a sense of responsibility and purpose.

The third domain comprised items from the previously published FACToR scale. The published item loadings did not fit our data as well as the alternative model determined by the CFA which we chose to use instead in this analysis. The alternative model identified four factors (CFI = 0.78, TLI = 0.68) which we named negativity and uncertainty (six items), positive emotions (two items), positive outlook (two items), and privacy concerns (two items). Negativity and uncertainty include emotional responses such as being upset, anxious, sad, or frustrated as well as uncertainty about test results and the implications for family. Positive emotions include feeling happy or relieved about genetic test results. Positive outlook reflects feeling that genetic testing helped clarify future disease prevention and other healthcare planning. The fourth scale, privacy concerns, relates to worry that genetic testing results would affect health insurance or employment status.

### 3.1 Scale correlates

Barrier subscale scores (Table 3), Motivators subscale scores (Table 4), and FACToR subscale scores (Table 5) are shown for each group described below.

**TABLE 3:** Barrier MIMIC

	Emotional concerns		Insurance		Social and interpersonal concerns		Family worry	
Variable	Est	*p*‐Value	Est	*p*‐Value	Est	*p*‐Value	Est	*p*‐Value
Sex (female vs. male)	0.801	0.001	0.4	0.041	0.396	0.086	0.527	0.04
Age	−0.337	<0.001	−0.1	0.126	−0.192	0.019	−0.211	0.04
Children (yes vs. no)	0.285	0.12	−0.181	0.198	0.14	0.437	0.86	<0.001
Years since diagnosis	0.103	0.048	0.157	0.014	0.046	0.447	0.068	0.261
BRCA‐associated cancer (yes vs. no)	0.345	0.237	0.138	0.419	0.548	0.005	0.253	0.303
Lynch‐associated cancer (yes vs. no)	−0.06	0.73	0.018	0.893	0.176	0.303	−0.048	0.811
Anxiety (any vs. none)	−0.435	0.158	−0.437	0.075	−0.198	0.634	−0.276	0.446
Depression (any vs. none)	0.938	<0.001	0.375	0.07	1.018	<0.001	0.738	0.005

**TABLE 4:** Motivator MIMIC

	Internally motivated emotions		Health benefits		Interpersonal emotions	
Variable	Est	*p*‐Value	Est	*p*‐Value	Est	*p*‐Value
Sex (female vs. male)	0.188	0.668	0.932	0.013	0.153	0.669
Age	−0.005	0.99	−0.122	0.28	0.078	0.588
Children (yes vs. no)	0.013	0.99	−0.002	0.99	0.073	0.828
Years since diagnosis	0.158	0.181	0.135	0.181	0.088	0.413
BRCA‐associated cancer (yes vs. no)	−0.234	0.588	−0.134	0.669	−0.045	0.99
Lynch‐associated cancer (yes vs. no)	−0.298	0.266	−0.023	0.99	−0.197	0.508
Anxiety (any vs. none)	−0.534	0.266	−0.472	0.413	−0.523	0.266
Depression (any vs. none)	−0.265	0.508	0.157	0.668	0.076	0.896

**TABLE 5:** FACToR MIMIC

	Neg/uncertainty		Positive emotions		Positive outlook		Privacy concerns	
Variable	Est	*p*‐Value	Est	*p*‐Value	Est	*p*‐Value	Est	*p*‐Value
Sex (female vs. male)	0.328	0.18	0.012	0.953	0.875	0.003	0.344	0.18
Age	−0.042	0.884	0.053	0.884	0.008	0.945	−0.08	0.616
Children (yes vs. no)	0.22	0.442	−0.032	0.945	0.196	0.495	−0.087	0.93
Years since diagnosis	−0.006	0.945	0.024	0.93	0.07	0.442	0.021	0.945
BRCA‐associated cancer (yes vs. no)	0.265	0.442	0.22	0.683	−0.036	0.945	0.365	0.241
Lynch‐associated cancer (yes vs. no)	0.071	0.93	0.112	0.884	0.066	0.93	0.212	0.477
Anxiety (any vs. none)	0.047	0.945	0.046	0.945	−0.178	0.884	−0.261	0.884
Depression (any vs. none)	0.984	0.003	−0.089	0.93	−0.024	0.945	0.664	0.18

### 3.2 Sex

Female respondents had significantly higher scores than males on three of the four barrier scales: emotional concerns, insurance concerns, and family concerns. Female patients also had significantly higher scores on the healthcare benefits motivator scale and the positive outlook scale of the FACToR domain than males. These results support hypothesis one regarding family concerns but did not support the anticipated increase in social and interpersonal concerns. We did not have a priori hypotheses regarding health benefits motivator scale nor the positive outlook FACToR scale.

### 3.3 Age

Younger age was associated with significantly higher scores on three of the four barrier scales, emotional concerns, social and interpersonal concerns, and family concerns. Age was not associated with any of the motivator or FACToR scales.

### 3.4 Having children

Having children was associated with significantly higher scores on one barrier scale, family concerns, but was not associated with any other scales. This result corresponds to hypothesis three.

### 3.5 Time from diagnosis

Further time from cancer diagnosis was significantly associated with increased concern about insurance implications of genetic testing. Further time from cancer diagnosis was also significantly associated with increased emotional concerns scale. Time since diagnosis was not associated with any motivator or FACToR scales.

### 3.6 Cancer type

Having a BRCA‐related cancer type was significantly associated only with higher scores on the social and interpersonal concern scale compared to non‐BRCA‐related cancer types. Having a Lynch‐associated cancer was not associated with any of the scales. Hypothesis four related to the social and interpersonal concerns scale result but the results did not support the health benefits part of the hypothesis.

### 3.7 Mental health

Having a mild‐to‐moderate depression score on the PHQ‐4 was associated with significantly higher scores for three of the four barrier scales (emotional concerns, social and interpersonal concerns, and family concerns) as well as the FACToR scale of negativity/uncertainty compared to those without depression. Presence of anxiety was not associated with any of the scales.

## 4 DISCUSSION

This cross‐sectional study evaluated barriers and motivators to genetic testing among a previously tested population as a pilot study for a larger interventional clinical trial. Patient‐level barriers to genetic testing include emotional, insurance, social and interpersonal, and family concerns. Several demographic characteristics (female, younger age, having children, longer time since diagnosis, BRCA‐associated cancer type, and depression) were associated with increase report of barriers to genetic testing. Patient‐level motivators for genetic testing include internally motivating emotions, health benefits, and interpersonal emotions. Female participants recalled being motivated by the health benefits more than their male counterparts. No other demographic characteristics were associated with motivators.

Females had increased social and interpersonal concerns and family concerns regarding genetic testing compared to males. Studies have shown that women often report these concerns as barriers to testing however there is a lack of research regarding the psychosocial barriers to testing for men Given that males are nearly three times less likely to get testing than females, elucidating the barriers to testing for men is a high priority.^33^ By having both men and women in our study, we are able to show that there is a significant sex difference in barriers to genetic testing, despite the relatively small percentage of males in our cohort. Further research in a more balanced cohort of men and women is necessary to elucidate the barriers more strongly impacting the decision about genetic testing in men.

Being younger was associated with greater social and interpersonal and family concerns which are consistent with literature reporting increased concerns about family members in young patients receiving genetic testing.^34^ Younger respondents also expressed more negative emotions as well which has not been previously reported in the literature. While clinicians may suggest speaking to mental health professional regarding an early‐onset cancer diagnosis, referral to a genetic counselor may also be needed. Our findings suggest that placing a referral for genetic testing among patients dealing with depression without further psychological support is unlikely to lead to testing.

Having a diagnosis of BRCA‐associated cancer was associated with greater social and interpersonal concerns. This is consistent with studies reporting barriers to testing related to the response of people other than the proband.^35^, ^36^ Addressing these barriers, as well as providing resources that facilitate the conversation with family members about genetic testing results should be investigated.

A striking finding was that self‐reported depressive symptoms were associated with higher report of retrospective barriers for three of the four subscales as well as the negativity/uncertainty subscale of the FACToR. Lerman et al. in 1999 showed that depressive symptoms were a barrier to uptake of genetic testing for Lynch Syndrome patients but there has been a lack of more recent studies evaluating the impact of depression on genetic testing uptake.^37^ These findings have implications for both pretesting and post‐testing counseling. Providers may need to proactively address barriers among those with a history of depression to help them overcome their resistance or ambivalence to genetic testing. Our findings suggest that placing a referral for a patient with depression without further discussion covering barriers and motivators is likely to result in that patient not making an appointment and getting the testing that is clinically indicated. With regard to post‐test counseling, the association of depression with the negativity/uncertainty FACToR subscale suggests that clinicians may want to take extra care to prepare patients with a history of depression in the genetic testing referral process as well as discussion of their genetic testing results.

There were several findings for which we had no a priori hypotheses. For example, being female was also associated with increased insurance concerns, a stronger interest in health benefits, and increased positive outlook on the FACToR scale. Given that the majority of the female respondents had breast cancer (62.5%), the latter two findings may be related to greater public awareness of the availability and benefits of genetic testing for breast cancer, however, as we did not ask about this specifically, there may be other underlying reasons for this result.^38^ We also found that having a more distant cancer diagnosis was associated with increased insurance concerns and greater emotional concerns. This may be explained by the fact that participants who are receiving or have recently completed treatment are less concerned with factors such as future insurance coverage and emotional concerns as they are more preoccupied with more immediate heath concerns such as access to care and treatment costs.^39^, ^40^ Further research is needed to understand the reasons why these barriers and motivators were significant for respondents and what role they played in testing decisions. In particular, follow‐up surveys for respondents who had a stronger interest in health benefits and had a more positive outlook after testing would guide clinicians in their discussions of testing decisions.

Overall, motivators were not associated with any demographic or clinical variables. The one exception was that females were more likely than males to report an association with anticipated health benefits of testing. Further research is needed to elucidate motivators for genetic testing. Potential future studies include qualitative studies with a demographically diverse patient cohort who have completed genetic testing. We propose that the reasons individuals are motivated to pursue genetic testing may be particularly personal and current research has not captured them appropriately.

The study has several limitations. First is the retrospective manner in which the barrier and motivator questions were assessed. We queried participants to report what they recalled feeling prior to genetic testing for the barrier and motivator questions. It is possible that the experience of getting tested or the timing of their testing (months, years ago) may have impacted their recall. Future prospective studies surveying participants before undergoing genetic testing are needed. Another area to address in future work Is expanding our awareness of additional barriers and motivators. While our survey was informed by literature review and existing measures, it is possible that patients had other barriers/motivators to testing that were not listed in our survey. Another limitation is that the sample population was largely homogeneous; predominantly female (92.5%), white (90.0%), and had BRCA‐related cancer (89.2%). Exploring our findings in more demographically and clinically diverse sample is mandated. The data from this study are part of a pilot project and the associated clinical trial is underway with efforts to reach underrepresented populations by enrolling participants from a wide variety of clinical sites across the state of Michigan which serve a more diverse population than the medical center where this study was performed. As a part of this larger clinical trial, we will be gathering information on barriers and motivators as well as testing recommendations and whether or not participants received genetic testing allowing us to further elucidate the correlations between testing status and barriers and motivators.

## AUTHOR CONTRIBUTIONS

**Erika N. Hanson:** Conceptualization (equal); data curation (supporting); project administration (equal); writing – original draft (lead); writing – review and editing (lead). **Emerson Delacroix:** Project administration (equal); writing – review and editing (equal). **Sarah Elizabeth Austin:** Writing – review and editing (equal). **Grant Carr:** Formal analysis (equal); writing – review and editing (supporting). **Kelley M Kidwell:** Formal analysis (equal); writing – review and editing (supporting). **Elizabeth Bacon:** Data curation (equal); project administration (supporting); writing – review and editing (supporting). **Lynette Hammond Gerido:** Writing – review and editing (supporting). **Jennifer J Griggs:** Funding acquisition (equal); writing – review and editing (equal). **Elena M. Stoffel:** Funding acquisition (equal); methodology (supporting); writing – review and editing (equal). **Ken Resnicow:** Conceptualization (equal); funding acquisition (equal); methodology (equal); supervision (equal); writing – review and editing (equal).

## FUNDING INFORMATION

NIH/NCI U01 CA232827‐05: Innovative Approaches to Expand Cancer Genetic Screening and Testing for Patients & Families in a Statewide Oncology Network through Community, State, & Payer Partnerships (PI Stoffel, Griggs, Resnicow).

## CONFLICT OF INTEREST STATEMENT

None of the authors have a conflict of interest to disclose.

## Data Availability

The data that support the findings of this study are available from the corresponding author upon reasonable request.

## References

[1] Walker EJ , Carnevale J , Pedley C , et al. Referral frequency, attrition rate, and outcomes of germline testing in patients with pancreatic adenocarcinoma. Fam Cancer. 2019;18(2):241‐251. 30267352 10.1007/s10689-018-0106-2

[2] Suri Y , Yasmeh JP , Basu A . Understanding the uptake and challenges of genetic testing guidelines for prostate cancer patients. Cancer Treat Res Commun. 2022;32:100588. 35759831 10.1016/j.ctarc.2022.100588

[3] Childers CP , Childers KK , Maggard‐Gibbons M , Macinko J . National estimates of genetic testing in women with a history of breast or ovarian cancer. J Clin Oncol. 2017;35(34):3800‐3806. 28820644 10.1200/JCO.2017.73.6314 PMC5707208

[4] Frey MK , Finch A , Kulkarni A , Akbari MR , Chapman‐Davis E . Genetic testing for all: overcoming disparities in ovarian cancer genetic testing. Am Soc Clin Oncol Educ Book. 2022;42:1‐12. 10.1200/EDBK_350292 35452249

[5] Wehbe A , Manning M , Assad H , Purrington KS , Simon MS . Uptake of genetic counseling and testing in a clinic‐based population of women with breast cancer. Cancer Med. 2022;11(17):3304‐3311. 35322585 10.1002/cam4.4684 PMC9468430

[6] Actkins KV , Srinivasan S , Spees LP , Turbitt E , Allen CG , Roberts MC . Uptake of genetic testing among patients with cancer at risk for lynch syndrome in the National Health Interview Survey. Cancer Prev Res (Phila). 2021;14(10):927‐932. 34341014 10.1158/1940-6207.CAPR-21-0073 PMC8492535

[7] Moretz C , Byfield SDC , Hatchell KE , et al. Comparison of germline genetic testing before and after a medical policy covering universal testing among patients with colorectal cancer. JAMA Netw Open. 2022;5(10):e2238167. 36279135 10.1001/jamanetworkopen.2022.38167 PMC9593236

[8] Smith‐Uffen M , Bartley N , Davies G , Best M . Motivations and barriers to pursue cancer genomic testing: a systematic review. Patient Educ Couns. 2021;104(6):1325‐1334. 33390305 10.1016/j.pec.2020.12.024

[9] Muessig KR , Zepp JM , Keast E , et al. Retrospective assessment of barriers and access to genetic services for hereditary cancer syndromes in an integrated health care delivery system. Hered Cancer Clin Pract. 2022;20(1):7. 35144679 10.1186/s13053-022-00213-5 PMC8832647

[10] Mallen AR , Conley CC , Fuzzell L , et al. "I think that a brief conversation from their provider can go a very long way": patient and provider perspectives on barriers and facilitators of genetic testing after ovarian cancer. Support Care Cancer. 2021;29(5):2663‐2677. 32975643 10.1007/s00520-020-05779-1 PMC7981241

[11] Hurtado‐de‐Mendoza A , Graves K , Gómez‐Trillos S , et al. Provider's perceptions of barriers and facilitators for Latinas to participate in genetic cancer risk assessment for hereditary breast and ovarian cancer. Healthcare (Basel). 2018;6(3):116. 30227649 10.3390/healthcare6030116 PMC6164735

[12] Czekalski MA , Huziak RC , Durst AL , Taylor S , Mai PL . Mainstreaming genetic testing for epithelial ovarian cancer by oncology providers: a survey of current practice. JCO Precis Oncol. 2022;6:e2100409. 35025618 10.1200/PO.21.00409

[13] Prochniak CF , Martin LJ , Miller EM , Knapke SC . Barriers to and motivations for physician referral of patients to cancer genetics clinics. J Genet Couns. 2012;21(2):305‐325. 21842318 10.1007/s10897-011-9401-x

[14] Paller CJ , Antonarakis ES , Beer TM , et al. Germline genetic testing in advanced prostate cancer; practices and barriers: survey results from the germline genetics working Group of the Prostate Cancer Clinical Trials Consortium. Clin Genitourin Cancer. 2019;17(4):275‐282 e1. 31171481 10.1016/j.clgc.2019.04.013 PMC6662206

[15] Loeb S , Li R , Sanchez Nolasco T , et al. Barriers and facilitators of germline genetic evaluation for prostate cancer. Prostate. 2021;81(11):754‐764. 34057231 10.1002/pros.24172

[16] Timbie JW , Kranz AM , Mahmud A , Damberg CL . Specialty care access for Medicaid enrollees in expansion states. Am J Manag Care. 2019;25(3):e83‐e87. 30875176 PMC6986199

[17] Mouslim MC , Johnson RM , Dean LT . Healthcare system distrust and the breast cancer continuum of care. Breast Cancer Res Treat. 2020;180(1):33‐44. 31983018 10.1007/s10549-020-05538-0 PMC7675785

[18] Bélisle‐Pipon JC , Vayena E , Green RC , Cohen IG . Genetic testing, insurance discrimination and medical research: what the United States can learn from peer countries. Nat Med. 2019;25(8):1198‐1204. 31388181 10.1038/s41591-019-0534-z

[19] Helmes AW . Application of the protection motivation theory to genetic testing for breast cancer risk. Prev Med. 2002;35(5):453‐462. 12431894 10.1006/pmed.2002.1110

[20] Scott D , Friedman S , Telli ML , Kurian AW . Decision making about genetic testing among women with a personal and family history of breast cancer. JCO Oncol Pract. 2020;16(1):e37‐e55. 31613719 10.1200/JOP.19.00221

[21] Missiha SB , Solish N , From L . Characterizing anxiety in melanoma patients. J Cutan Med Surg. 2003;7(6):443‐448. 15926214 10.1177/120347540300700602

[22] Niedzwiedz CL , Knifton L , Robb KA , Katikireddi SV , Smith DJ . Depression and anxiety among people living with and beyond cancer: a growing clinical and research priority. BMC Cancer. 2019;19(1):943. 31604468 10.1186/s12885-019-6181-4 PMC6788022

[23] Bergenmar M , Nilsson B , Hansson J , Brandberg Y . Anxiety and depressive symptoms measured by the hospital anxiety and depression scale as predictors of time to recurrence in localized cutaneous melanoma. Acta Oncol. 2004;43(2):161‐168. 15163164 10.1080/02841860310021518

[24] Mella S , Muzzatti B , Dolcetti R , Annunziata MA . Emotional impact on the results of BRCA1 and BRCA2 genetic test: an observational retrospective study. Hered Cancer Clin Pract. 2017;15:16. 29026449 10.1186/s13053-017-0077-6 PMC5625658

[25] Kroenke K , Spitzer RL , Williams JB , Löwe B . An ultra‐brief screening scale for anxiety and depression: the PHQ‐4. Psychosomatics. 2009;50(6):613‐621. 19996233 10.1176/appi.psy.50.6.613

[26] Fogleman AJ , Zahnd WE , Lipka AE , et al. Knowledge, attitudes, and perceived barriers towards genetic testing across three rural Illinois communities. J Community Genet. 2019;10(3):417‐423. 30673953 10.1007/s12687-019-00407-w PMC6591342

[27] Hafertepen L , Pastorino A , Morman N , et al. Barriers to genetic testing in newly diagnosed breast cancer patients: do surgeons limit testing? Am J Surg. 2017;214(1):105‐110. 27773374 10.1016/j.amjsurg.2016.08.012

[28] Shaw J , Bulsara C , Cohen PA , et al. Investigating barriers to genetic counseling and germline mutation testing in women with suspected hereditary breast and ovarian cancer syndrome and lynch syndrome. Patient Educ Couns. 2018;101(5):938‐944. 29273311 10.1016/j.pec.2017.12.011

[29] Gill G , Beard C , Storey K , Taylor S , Sexton A . "It wasn't just for me": motivations and implications of genetic testing for women at a low risk of hereditary breast and ovarian cancer syndrome. Psychooncology. 2020;29(8):1303‐1311. 32497346 10.1002/pon.5436

[30] Fogel AL , Jaju PD , Li S , Halpern‐Felsher B , Tang JY , Sarin KY . Factors influencing and modifying the decision to pursue genetic testing for skin cancer risk. J Am Acad Dermatol. 2017;76(5):829‐835 e1. 28087134 10.1016/j.jaad.2016.11.050

[31] O'Neill SC , Lipkus IM , Sanderson SC , Shepperd J , Docherty S , McBride CM . Motivations for genetic testing for lung cancer risk among young smokers. Tob Control. 2013;22(6):406‐411. 22744911 10.1136/tobaccocontrol-2011-050306 PMC3586780

[32] Li M , Bennette CS , Amendola LM , et al. The feelings about genomiC testing results (FACToR) questionnaire: development and preliminary validation. J Genet Couns. 2019;28(2):477‐490. 30964586 10.1007/s10897-018-0286-9

[33] Childers KK , Maggard‐Gibbons M , Macinko J , Childers CP . National Distribution of cancer genetic testing in the United States: evidence for a gender disparity in hereditary breast and ovarian cancer. JAMA Oncol. 2018;4(6):876‐879. 29710084 10.1001/jamaoncol.2018.0340 PMC6145682

[34] Morand M , Roth M , Peterson SK , et al. Factors impacting adolescent and young adult cancer patients' decision to pursue genetic counseling and testing. Support Care Cancer. 2022;30(6):5481‐5489. 35306607 10.1007/s00520-022-06974-y PMC9703615

[35] Schlich‐Bakker KJ , Ten Kroode HFJ , Wárlám‐Rodenhuis CC , van den Bout J , Ausems MGEM . Barriers to participating in genetic counseling and BRCA testing during primary treatment for breast cancer. Genet Med. 2007;9(11):766‐777. 18007146 10.1097/gim.0b013e318159a318

[36] Zimmermann BM , Fanderl J , Koné I , et al. Examining information‐seeking behavior in genetic testing for cancer predisposition: a qualitative interview study. Patient Educ Couns. 2021;104(2):257‐264. 32988685 10.1016/j.pec.2020.09.019

[37] Lerman C et al. Genetic testing in families with hereditary nonpolyposis colon cancer. JAMA. 1999;281(17):1618‐1622. 10235155 10.1001/jama.281.17.1618

[38] Giri VN , Shimada A , Leader AE . Predictors of population awareness of cancer genetic tests: implications for enhancing equity in engaging in cancer prevention and precision medicine. JCO Precis Oncologia. 2021;5:1699‐1708. 10.1200/PO.21.00231 PMC8585288 34778693

[39] Stump TK , Eghan N , Egleston BL , et al. Cost concerns of patients with cancer. J Oncol Pract. 2013;9(5):251‐257. 23943901 10.1200/JOP.2013.000929 PMC3770507

[40] Paul C et al. Cancer patients' concerns regarding access to cancer care: perceived impact of waiting times along the diagnosis and treatment journey. Eur J Cancer Care (Engl). 2012;21(3):321‐329. 22111696 10.1111/j.1365-2354.2011.01311.x PMC3410528

